# Pre-elimination of malaria on the island of Príncipe

**DOI:** 10.1186/1475-2875-9-26

**Published:** 2010-01-20

**Authors:** Pei-Wen Lee, Chia-Tai Liu, Herodes Sacramento Rampao, Virgilio E do Rosario, Men-Fang Shaio

**Affiliations:** 1 The Anti-Malaria Team of Taiwan in São Tomé and Príncipe, São Tomé and Princípe; 2 Taiwan Urbani Foundation, Taipei, Taiwan; 3 Centro National de Endemias, São Tomé, Democratic Republic of São Tomé and Príncipe; 4 Centro de Malaria e Doencas Tropicais/Instituto de Higiene e Medicina Tropical/Universidade Nova de Lisboa, Lisbon, Portugal; 5 Institute of Clinical Nutrition/HungKuang University, Sal-Lu, Taichung, Taiwan

## Abstract

**Background:**

*Plasmodium falciparum *is the major species responsible for malaria transmission on the island of Príncipe, in the Republic of São Tomé and Príncipe (STP). Indoor residual spraying (IRS) has been intensively deployed on the island, since 2003. Other measures included intermittent preventive therapy (IPT), since 2004, as well as artemisinin-based therapy (ACT) and long-lasting insecticidal nets (LLINs) from 2005. The work was coordinated by the Ministry of Health of STP through their Centro Nacional de Endemias (CNE) and the impact of such an integrated control programme on the prevalence and epidemiology of malaria in Príncipe was evaluated.

**Methods:**

The scaling-up of preventive strategies included IRS, LLINs, IPT for pregnant women, as well as early diagnosis and prompt treatment with ACT. Regular implementation of an island-wide IRS programme was carried out yearly in 2003-2005, and later in 2008. Malaria incidence and prevalence were estimated based on passive case detection and active case detection, respectively. Slide positivity rate (SPR) was used as an indicator of any increase of malaria cases during and after the control programme was initiated.

**Results:**

Regular IRS achieved a coverage of 85-90% for each of the four annual cycles (2003-2005, annually and one spraying in 2008) while usage of LLINs was never superior to 50% from 2006-2009. Coverage of IPT steadily increased from 50% in 2004 to 80% in 2008. Since 2006, over 90% of uncomplicated malaria patients received ACT treatment. Severe malaria cases were hospitalized and treated with quinine. Monthly trends of SPR were constantly over 50% in 2003, but steadily decreased below 10% in 2006. SPR has been below 5% since 2007, but an increase to up to 15% was noted in June 2009 when 16 imported cases were detected. A steep decline by 99% of malaria incidence was observed between 2003 and 2008, with an incidence risk of the population of five per thousand, in 2008. No malaria mortality has been reported since 2005. Species shift from falciparum to non-falciparum malaria was noted after a five-year intensive control programme. Cross-sectional country-wide active surveillances showed malaria prevalences of 1.1%, 0.7%, and 0.9% in June 2006, Oct 2007, and July 2009, respectively, of which over 90% were asymptomatic.

**Conclusion:**

The effective measures of the combination of four major control methods have produced a rapid decline in malaria morbidity and mortality on the island of Príncipe. The combination of IRS, IPT, and active surveillance with ACT treatment seemed to have played important roles to achieve a present status of low and stable malaria on the island. In low transmission settings, any increase of malaria morbidity indicates potential epidemics and assumes that current control strategies were interrupted. Active surveillance should be reinforced to follow and monitor all asymptomatic carriers and imported cases. Consolidation and a shift to elimination phase demands the sustainability of such integrated programmes.

## Background

The island of Príncipe, isolated in the Gulf of Guinea, is the smaller partner island in the archipelago of São Tomé and Príncipe (STP). Falciparum malaria was considered the most important vector-borne disease on the island of Príncipe with a prevalence of 35% of the population exhibiting parasitaemia, as reported in 1997 [1]. A reduction of prevalence to 0.6% was noted in the early 1980s, after an eradication programme was carried out using mainly indoor residual spraying (IRS) with dichlorodiphenyltrichloroethane (DDT) and weekly prophylaxis with chloroquine [2]. However, the eradication programme was disrupted in 1982, due to financial difficulties and lack of a long-lasting control programme. The rebound of malaria with high mortality in small children was observed in 1984 [2]. The situation worsened with the emergence of mosquito resistance to DDT and the appearance of *Plasmodium falciparum *chloroquine-resistant strains. Since then, malaria became a major public health problem until 2003, when the Taiwan International Cooperation and Development Fund (ICDF) collaborated with the São Tomean Government to implement a nationwide indoor residual spraying programme to reduce the presence of *Anopheles *mosquitoes [3]. Of the two islands of STP, Príncipe was selected as the pilot place for IRS in 2003 [3]. The scaling-up of preventive strategies included yearly cycle of IRS with alphacypermethrin starting in 2004, followed by intermittent preventive therapy (IPT) in pregnant women with sulphadoxine-pyrimethamine (SP) in 2004, the widespread use of artemisinin-based combination therapy (ACT) in 2005, and the national long-lasting insecticidal nets (LLINs) campaign also in 2005 [4]. A remarkable reduction in malaria morbidity and mortality was observed after three yearly cycles of IRS [4,5]. Since 2007, malaria transmission has been constantly low and stable in Príncipe [5]. Current malaria control strategies are better than those applied in the 1980s, as they include both vector control strategies and case management. Further, the use of alphacypermethrin in IRS is still fully effective and ACT resistance has not been observed. However, dramatic decreases in morbidity and mortality may be short-lived if the current integrated control programme cannot be sustained. The low transmission settings could cause many people to lose immunity against the disease, rendering it more devastating if returned. Lessons from the resurgence of malaria in India and Sri Lanka have suggested that partial victory over malaria could be worse than total failure [6]. The previous 1980 failure in STP on attempts for malaria elimination, which was followed by major mortality, can be repeated now if sustainability of the programme is not maintained. With a unique isolated geographic position, and low number of inhabitants, the island of Príncipe is a place where malaria elimination may be feasible. A number of control strategies were applied in a coordinated effort, in a consolidated and sustained integrated programme for eliminating malaria from this island, investigating whenever possible, insufficiencies and difficulties.

## Methods

### Sites

The island of Príncipe with a total area of 142 km^2^, is part of the two island archipelago of STP, situated in the Gulf of Guinea, about 160 km north-east of the island of São Tomé. It is volcanic in origin, mountainous, with a long-lasting rain season and still largely covered by primary and secondary rainforest. According to the last census, it maintains ca. 6,000 inhabitants [7]. Most houses were wooden built with zinc-plated roofs.

The programme was initiated in 2003, and a laboratory was set in the main island of São Tomé, following STP government directives for malaria control and for ethical clearance throughout the implementation of the programme. Informed verbal consent was obtained from residents who answered a short questionnaire, which included information on the use of bed nets. Parents responded on behalf of infants and children.

### IRS

In 2003, STP government and Taiwan ICDF launched a long-term project on malaria elimination, where some of these strategies were studied and published [3]. In addition to a pilot IRS performed in September 2003, regular implementation of island-wide IRS programme was carried out three times, i.e., in October 2004, October 2005 and August 2008, respectively. The concentration of alphacypermethrin (Hockley International Ltd, Stockport, UK) sprayed on the wall surfaces throughout the four cycles was 50 mg per square meter [3]. It took two weeks to complete each cycle of island-wide IRS. The average acceptance rates of IRS for dwellings and outhouses were constantly over 90% for each of the three cycles during 2003-2005 and around 85% for the 4^th ^cycle in 2008. Visiting households and checking for mosquito mortality by cone bioassays was carried out regularly and labeling of houses under IRS treatment was also carried out. To evaluate residual activity of alphacypermethrin, standard cone bioassays were undertaken bimonthly according to previously report [8]. This programme was complemented with LLIN, in 2005, and larviciding in 2007.

### LLINs

Since 2005, LLINs have been distributed freely to children and pregnant women (with funding from many international donors such as the Global Fund against AIDS, Tuberculosis and Malaria (GFATM) covering the entire island). Although an LLINs ownership rate of two nets per household was >80% [9], the usage rate of LLINs for residents on the island was constantly less than 50% over the year 2006-2009 (results obtained by using questionnaire when active surveillance and IRS were carried out).

### Larviciding

Young mosquito larvae detected during surveillance operations were treated with *Bacillus thuringiensis israelensis *(Bti, VectoBac G, Lot number 145-077-N8, 200 ITU/mg, Valent Bioscience Corporation, Libertyville, Ill), which was regularly applied (once per week) to permanent breeding sites through the whole year. In the island, seven breeding sites were identified and these stayed active during the dry season. A team of three local technicians was provided with Bti using the dose of 1 gram/m2. Temporary breeding sites found along the roadside during the rainy season were also examined and treated. For the determination of the quantity of larvae in the breeding sites, a standard dipper of 250 ml was used [10]. A pre-determined number of dips (60 ml) was taken from different sites within each breeding site, usually ponds, and the average number of mosquito larvae registered. Mosquito species identification was carried out at a later stage. The percentage reduction in larval mosquito densities was calculated using the formula of Mulla *et al *[11]. The killing effect of Bti on young larvae was evaluated as previously reported [12,13] and Bti applied again to those breeding sites where larvae survived.

### IPT

Since 2004, pregnant women received sulphadoxine and pyrimethamine (SP) for IPT during the 4^th ^and 7^th ^month of pregnancy [14]. Coverage steadily increased from 50% in 2004 to 80% in 2008 [15]. This was carried out in the health centers under clinicians or nurses supervision.

### ACT

With support from GFATM, artesunate-amodiaquine has become the first-line treatment for uncomplicated malaria since 2005. Artemether-lumefantrine (Coartem^®^, Novartis) is currently used as the second-line drug for malaria treatment. Uncomplicated malaria patients diagnosed by passive case detection or active case detection received ACT treatment or were admitted to hospital for quinine treatment when showing severe symptoms. Women suffering from malaria during their first trimester of pregnancy were also treated with quinine. Since 2006, over 90% of uncomplicated malaria patients received ACT treatment [16].

### Malaria survey and management

Malaria is diagnosed through passive case detection by using optical microcopy in hospitals and the district health centers and through mass screening by use of the rapid diagnostic tests (RDTs, ICT Diagnostics). To verify both the sensitivity and specificity of RDTs, in 2005, 304 cases of fever (body temperature > 37.5°C) were tested by both RDT and blood film examination. The discrepancy in results from these two methods was further clarified by polymerase chain reaction (PCR) [1,17], carried out locally. Because PCR is demonstrably more sensitive than microscopy, a positive finding by PCR in parallel with a negative microscopy but positive RDT was regarded as positive. Therefore, malaria positive cases were defined as either microscopic positive or/and PCR positive. Malaria positive cases received ACT treatment except that severe malaria and pregnant women (first trimester of pregnancy) were given quinine intravenously, according to local regulations.

Data collected from the hospital and health unities in Principe was sent weekly to the Taiwan anti-malaria team in the main island.

### Passive case detection

The daily records of malaria detection in health stations were reviewed and complied in order to calculate the *P. falciparum *incidence and slide positivity rate (SPR). Microscopic reading on blood films was performed according to CNE diagnostic protocols. Throughout the control programme during 2003-2009, microscopic examination has been used as the gold standard method. Two trained microscopists examined the blood films simultaneously and a third one clarified any discrepancy in results. A blood film was declared negative when no parasite was detected in 200 fields. Once malaria patients were diagnosed by passive case detection, within one week, members of the patient family in the same house and residents in adjacent houses were asked to supply a blood sample for microscopy examination.

### Mass screening

Through a cross-sectional island-wide survey, active surveillance (mass screening) by use of RDTs was carried out three times, i.e., in June 2006, October 2007, and July 2009. Internal quality control of RDTs included an immediate blind second reading of 100% of the RDTs. In case of disagreement, two technicians re-examined the RDT together and decided on the reading. All positive cases found by RDTs were examined by optical microscopy of Giemsa-stained blood smears. The technician recording the microscopic result was unaware of the corresponding RDT results. Positive results by RDTs but negative by microscopic examination were further clarified by PCR. It took 10 days to complete an island-wide surveillance.

### Follow-up, evaluation, and monitoring

Patients found positive for malaria infection, either by passive case detection or by mass screening, were given anti-malaria treatment according to CNE guidelines. A registration card was filled in with the patient's name, sex, age, weight, body temperature, parasitaemia, drug regimen, and address (village or locality) was kept for follow-up. An uncomplicated malaria case was defined with a blood count of *P. falciparum *asexual parasitaemia > 0 parasites/μl, and not fulfilling the criteria for severe malaria, by microscopy or a positive RDT.

Severe malaria was defined as a malaria case with at least one of the following criteria: body temperature > 39°C, packed cell volume < 15%, prostration (inability to sit unaided or non-ambulant), vomiting or diarrhoea, impaired consciousness, hypoglycaemia, convulsions, respiratory distress (deep breathing or indrawing). Patients with severe malaria were treated with quinine intravenously. Treatment was switched to oral as soon as the patient was able to tolerate it. The uncomplicated malaria cases treated with ACT at home, were followed up by a mobile team (consisting of a nurse and a technician), which actively visited patients by taking blood films for microscopic examination two weeks after the three-day treatment. Treatment was repeated if parasitaemia persisted.

### Data analysis

Statistical analyses were performed using SPSS version 17.0. The annual trends of morbidity for the classes "<5 years old", "≥5 years old", and "pregnancy", were tested by Poisson regression for the years 2003 through 2009. The association of IRS and LLINs on malaria block transmission was measured through logistic regression analysis.

## Results

### Effect of vector controls

Cone bioassay showed that the alphacypermethrin steadily maintained its residual efficacy for 12 months when it was applied to wood walls but its insecticidal effect only persisted up to six months when applied to cement walls. This long-lasting residual effect of alphacypermethrin has been constantly observed since 2004. Larviciding assay showed that 100% mortality of young larvae was achieved after 24-hr Bti exposure at all permanent breeding sites during the dry season, but varied greatly from 30% to 80% during the rainy season.

An interview with 5,609 inhabitants carried out in July 2009 showed that during the first half year of 2009, unprotected residents were 273 (of which seven had malaria), 2,811 residents were under IRS programme (of which 21 had malaria), 228 under LLINs (of which five had malaria), and 2,297 under both IRS and LLINs (of which 19 had malaria). The results of the logistic regression analysis in Table 1 show that the combined use of IRS and LLINs has no additional protective effect against malaria when compared to the use of IRS alone (OR = 1.108, 95% CI: 0.594-2.066, p-value = 0.747 > 0.05). Being unprotected increases the odds by 3.5 (OR = 3.496, 95% CI: 1.473-8.300, p-value = 0.005) of that for IRS protection alone, while using LLINs alone, when compared with IRS protection alone, increased the odds by almost three-times (OR = 2.979, 95% CI: 1.113-7.975, p-value = 0.030).

**Table 1:** Effect of combining indoor residual spraying (IRS) and long-lasting insecticide-treated nets (LLINs) interventions on malaria infection in Príncipe for the year 2009

Intervention	Malaria cases	Total cases	OR	95% CI	P value
IRS only	21	2811	1		0.008
Unprotected	7	273	3.496	1.473 - 8.300	0.005
LLINs only	5	228	2.979	1.113 - 7.975	0.030
IRS + LLINs	19	2297	1.108	0.594 - 2.066	0.747

IRS: indoor residual spraying; LLINs: long lasting insecticide-treated nets;

OR: odds ratio; IC: confidence interval.

Taking together, 2,525 residents (of which 24 had malaria) frequently used LLINs and 5,108 residents slept (of which 40 had malaria) in IRS-treated houses (Table 2). Table 2 gives the results of the logistic regression unadjusted and adjusted for the variables IRS and LLINs. It can be seen that, whether a person had a bed net or not, living in an IRS-treated house gave a protective effect against malaria infection with an OR of 0.286 and a 95% CI of 0.12-0.679. The interaction between living in an IRS-treated house and having a bed net in the house seems not to be significant (OR = 1.301, 95% CI: 0.348-4.858, p-value = 0.696), meaning that there is no statistical evidence that living in an IRS treated house with a bed net has any additional protective effect against malaria infection. This is in accordance to the results on Table 1 where the OR of "IRS + LLINs" was not significantly different from the OR of "IRS alone".

**Table 2:** Effects of IRS, LLINs, unadjusted, and adjusted for each other on malaria infection in Príncipe for the year 2009

Intervention		Malaria cases	Total cases	OR	95% CI	P value	Adjusted OR	95% CI	P value
IRS	No	12	501	1					
	Yes	40	5108	0.322	0.168-0.617	0.001	0.286*	0.120-0.679	0.005

LLINs	No	28	3084	1					
	Yes	24	2525	1.047	0.606-1.811	0.869	0.852**	0.267-2.722	0.787

IRS + LLINs							1.301	0.348-4.858	0.696

RS: indoor residual spraying; LLINs: long lasting insecticide-treated nets; OR: odds ratio; IC: confidence interval.

*Adjusted for effect of LLINs.

**Adjusted for effect of IRS.

Whether a person lives in an IRS-treated house or not, bed net alone does not seem to have a protective effect against malaria infection. Although the adjusted OR is less than 1, it is not statistically significant (OR = 0.852, 95% CI: 0.267-2.722, p-value = 0.787). This is not surprising since the number of people with bed net alone is only 228, when compared with the total of 5,609 people in the study, of which almost all people (5,108) lived in IRS-treated houses.

### Validity tests of RDTs

Among 304 febrile subjects tested, 46 cases (15.1%) were positive and 258 cases were negative by RDT while 38 cases (12.5%) were positive and 266 cases were negative by blood film examination. Thirty-seven cases were positive by both RDT and blood film examination while 257 cases were negative by both RDT and blood film examination. One case was positive by blood film but negative by RDT and nine cases were negative by blood film but positive by RDT. Compared to blood film examination by microscopy (as gold standard), sensitivity and specificity of the RDT were 97.4% and 96.6%, respectively. While the positive predictive value of this RDT is 80.4%, its negative predictive value reaches 99.6%.

Throughout the study, optical microscopy was carried out for all passive case detection and for those positive cases by RDT in mass screening. With the moderate positive predictive value by RDTs, all positive cases by RDTs were examined microscopically. Since 2007, less than 100 subjects each year were tested by both RDT and blood films simultaneously. Confirmation of the data was carried with double blind reading by other trained microscopists. PCR was used whenever necessary. Through PCR we identified at the end of the programme a large prevalence of asymptomatic infections.

### Malaria incidence, SPR, and prevalence

**Figure 1:**
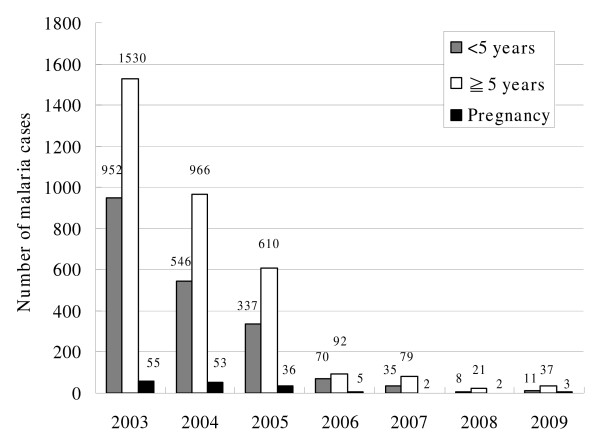
**Malaria morbidity on the island of Príncipe 2003-2009**.

Table 3 shows the decrease of malaria incidence from 2003, (>400 per 1,000 population) after the programme was implemented, with a 99% reduction in 2008. The remarkable decrease of malaria morbidity was noted in both age groups (Figure 1). Poisson regression was performed on malaria infection counts considering as factors the variables "Year" (2003-2009) and "AgeClass" (< 5 years, ≧5 years and pregnancy). Both main-effects terms (Year and AgeClass) were significant to the model (p-values < 0.001), and over-dispersion was not a problem for this data set (over-dispersion was checked by fitting a negative binomial model with ancillary parameter equal to 0 and by performing the Lagrange multiplier test, the p-value obtained was equal to 0.464).

**Table 3:** Malaria indicators on the island of Príncipe 2003-2009

Yeas	Population estimated	Positive cases by passive detection	Incidence (%)	Positive cases by mass screening	Prevalence (%)	Prevalence of malaria species**	Malaria mortality
2003	6157	2537	41.2	ND	ND	ND	7
2004	6255	1565	25.0	ND	ND	ND	10
2005	6355	983	15.5	ND	ND	ND	0
2006	6456	167	2.6	57	1.1	Pf: 53/Non-Pf: 4	0
2007	6558	116	1.8	37	0.7	Pf: 30/Non-Pf: 7	0
2008	6663	31	0.5	ND	ND	ND	0
2009*	6770	51	0.7	52	0.9	Pf: 30/Non-Pf: 22	0

*The 2009 incidence is reported for 11 months of the year for which data is available.

The population are projected from the 2001 census using 1.59% annual growth rate.

ND: not determined.

**Pf: infection with *Plasmodium falciparum*; Non-Pf: infection with any other three species of malaria (*P. vivax*, *P. ovale*, and *P. malariae*).

Table 4 shows that all the parameters are significant in the model since the p-values are all less than 0.001. This coefficient became more negative except for years 2008 and 2009 and there were fewer cases of malaria reported. Notice that despite the coefficient for year 2008 (-4.405) being smaller than the one for year 2009 (-3.907), the variability in 2008 is larger than 2009, clouding a strongest effect for 2008 to a certain extent (notice the overlap of the two 95% confidence intervals for these years). These results are confirmed through pairwise comparisons between years where, at 5% significance level, the only cases of non-significant differences are between the years 2008-2009 (*p*-value = 0.128) and 2006-2007 (*p*-value = 0.071). These exceptions, however did not affect the overall decreasing number of malaria infections since 2003. as there was no statistical difference in morbidity in these pairs of years.

**Table 4:** Parameter estimates by Poisson regression analysis

Parameter	Coefficient	Standard error	95% Wald CI	P value
(Intercept)	6.816	0.0386	6.740 - 6.891	< 0.001
Year 2009	-3.907	0.2034	-4.306 - -3.508	< 0.001
Year 2008	-4.405	0.2598	-4.914 - -3.895	< 0.001
Year 2007	-3.085	0.1365	-3.353 - -2.818	< 0.001
Year 2006	-2.721	0.1149	-2.946 - -2.496	< 0.001
Year 2005	-0.948	0.0540	-1.054 - -0.842	< 0.001
Year 2004	-0.483	0.0462	-0.574 - -0.393	< 0.001
Year 2003	0 ^a^			
Pregnancy	-2.530	0.1196	-2.765 - -2.296	< 0.001
≧ 5 years old	0.532	0.0409	0.452 - 0.612	< 0.001
<5 years old	0 ^a^			
(Scale)	2.068 ^b^			

Dependent Variable: malaria counts

Model: (intercept), Year, AgeClass

^a^Set to zero because this parameter is redundant.

^b ^Computed based on the Pearson chi-square.

Relatively to the age class factor, we see that, when comparing children of less than five years of age, to five years of age or older those contribute to an increase of malaria cases (coefficient = 0.532), while pregnancy (under IPT) contributed to an overall reduced number of malaria infections (coefficient = -2.530). These results are to be expected since, children under five years of age accounted for 35% - 40% of the total malaria cases before 2006 but this number was reduced to 20% afterwards (Figure 1). Also, the number of pregnant women with malaria decreased from 55 cases in 2003 to two and three cases in 2008 and 2009, respectively, corresponding to a reduction of malaria incidence in pregnant women of 96%. The pairwise comparisons were all significant (*p*-value < 0.001) for the three levels considered.

**Figure 2:**
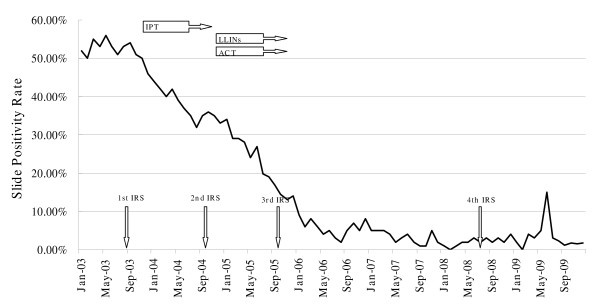
**Monthly trends of slide positivity rate (SPR) for malaria on island of Príncipe**. IRS: indoor residual spraying; IPT: intermittent preventive therapy; LLINs: long-lasting insecticidal nets; ACT: artemisinin-based therapy.

SPR of malaria episodes was over 50% in 2003 but a steady decline of SPR was seen afterwards (Figure 2). Monthly trends of SPR were reduced to 10% since 2006, with a decrease to below 5% from 2007 (Figure 2). In June 2009, an increase of SPR up to 15% was noted, which mainly due to imported cases from the main island of São Tomé. There were 31 and 51 malaria cases detected in 2008 and 2009, respectively, but in 2009, 16 cases were imported from São Tomé, which causes concern for the future, as these imported cases may affect the sources of malaria transmission. It must be stressed that few local cases were considered severe, in 2008 and 2009, and were treated with quinine and cured.

Three active surveillances were conducted in June 2006, October 2007, and July 2009, with positive rates of 1.1% (57/5427), 0.7% (37/5289), and 0.9% (52/5609), respectively. Of the positive cases, at the end of the programme, more than 90% were asymptomatic (neither history of fever in recent three days nor other clinical symptoms).

All positive cases found by mass screening were treated with ACT. Over 95% of the positive cases completed treatment and follow-up, of which over 95% became negative two weeks after the three-day treatment.

During the mass screening, falciparum malaria was found to account for 93% (53/57), 81% (30/37) and 58% (30/52) in 2006, 2007, and 2009, respectively (Table 3). Non-falciparum malaria infection was notably increased from 7% (4/57) in 2006 to 42% (22/52) in 2009. Of the 22 cases with non-falciparum malaria, there were 11, eight, and three cases for *Plasmodium malariae, Plasmodium vivax*, and *Plasmodium ovale*, respectively.

### Malaria mortality

Malaria mortality was more than 10 deaths per year before 2003. At the early stage of integrated malaria control programme, the majority (70%) of malaria deaths was in the children of less than five years of age, i.e., five and seven cases in 2003 and 2004, respectively. Malaria-specific deaths were defined as those with severe malaria admitted to hospital and died after treatment. No malaria mortality has been recorded since 2005 (Table 3).

## Discussion

The integrated malaria control programme has been intensively carried out on the island of Príncipe, since 2003, and produced very good results reaching a pre-elimination phase. The effective anti-malarial measures used (IRS, IPT and ACT) brought the island to the brink of malaria elimination. In addition to the regular IRS, high coverage with IPT and case management (both passive case detection and mass screening) with ACT treatment (all asymptomatic malaria cases received ACT treatment) contributed most to the low and stable malaria transmission in Príncipe. This work also showed that classical methods, for control, in islands produce good results, though monitoring and vigilance are essential.

Some achievements in malaria control on tropical islands have been reported elsewhere [18,19], though their results were not so impressive as in Príncipe. In Zanzibar (situated 40 Km off the coast of mainland Tanzania), malaria-associated morbidity and mortality decreased dramatically within two years following deployment of ACT alone and a further reduction of malaria parasite prevalence was noted after additional distribution of LLINs [18]. In Bioko (located 32 km off the coast of Cameroon), malaria transmission was decreased by IRS and case management [19] and there was strong evidence of a protective effect of IRS combined with LLINs relative to IRS alone [20]. These results, including ours, strongly suggest that the most effective malaria control programmes are those that apply a combination of tools.

The implementation of IRS which was initiated at an early stage of this programme, could have been the basis of such success in the reduction of both malaria morbidity and mortality. The role of LLINs, implemented at a later stage, with IRS, had no additional protective effect to block malaria transmission when compared to the use of IRS alone. LLINs alone have been demonstrated to reduce malaria morbidity and mortality in children in Kenya [21], but this effect was not observed in the Príncipe programme. This is not surprising since the number of people with LLINs alone is few (4%) when compared with the total of 5,609 people in the study, of which almost all people (91%) slept in IRS-treated houses. In low malaria transmission settings, some reports showed no incremental benefit associated with the use of LLINs in areas that had been IRS treated [22-24]. It has been proposed that high coverage with effective IRS had already reduced sporozoite rates to such a low level that nets were unable to make any additional impact [23]. Nevertheless, LLINs synergistic effect while associated to IRS on the blocking of malaria transmission has been observed elsewhere but further studies are necessary [20].

Before the effective intervention was taken, higher incidence of clinical and severe malaria was noted in under-five years of age children and at this stage a reduction of 40% down to 20% has been observed. This value should be further reduced with the continuation of the programme. In this island, malaria control had been achieved two decades earlier though it was short lived. Seventy percent of the malaria deaths were from this group in 2003 and 2004. It is known that acquired immunity with protection against clinical malaria can last for several years if regular exposure exists [25,26]. In low transmission settings, however, older children and adults may soon lose their semi-immunity status due to non-exposure to infection. Since 2005, no death attributed to malaria was registered, indicating good management of malaria cases.

With young children, pregnant women are also at higher risk of developing severe disease [27]. At the end of this control programme malaria incidence in pregnant women was lower than 1%. IPT had varying coverage, since its application in 2004, reaching 80% coverage in 2008 and no malaria mortality in pregnant women was detected since 2005. This result could be associated to any of the control measures used.

It was also observed, by mass screening, that asymptomatic malaria infections were present in high rates, among the positive cases. This is of relevance and both active and passive case detection of malaria, at community level, should be set up as crucial components of the elimination programme [28]. Health service delivery should be reinforced to shift from passive detection and case management to more active screening and systematic treatment/follow-up strategies, establishing a strong surveillance system [29]. Studies on this subject are strongly needed with both parasitological and clinical aspects.

*Plasmodium falciparum *was the predominant species, accounting for 90% of all cases of malaria on the island, before intervention was made [30] and was the main target in this control strategy. ACT alone can reduce the incidence and prevalence of malaria and is known to have an effect on the transmission of falciparum malaria [31]. Results from extensive surveillances indicate that a shift has been noted from falciparum malaria to non-falciparum malaria after the five-year intensive control programme, concerning mainly *P. vivax *and *P. malariae*. Relapse malaria is relevant because its control is more strongly dependent on treatment interventions than anti-vectorial measures [32]. The increase of *P. malaria *infection, which is normally suppressed by *P. falciparum*, can be due to the reduction in the prevalence of *P. falciparum *by the effective malaria control. A precedent for such a pattern change of infection and disease was observed in Tanzania during the 1950s [33].

At present, the hypoendemic status of malaria in Príncipe is similar to the 1980s' situation, with a dramatic reduction of morbidity and mortality after an intensive malaria control programme. Previous DDT eradication programme failed, mainly due to its discontinuation and the emergence of chloroquine-resistant *Plasmodium *parasites and DDT-resistant mosquitoes. Such failure and the consequent epidemics can happen again if current control programme is not sustainable. Although current control strategies are highly integrated, both treatment and mosquito control applied are vulnerable to the emergence of compound-resistant parasites and mosquitoes [34].

Concerning eventual set-backs to the control measures, neither resistance of mosquitoes to alphacypermethrin nor resistance of parasites to ACT has been found so far. Resistance to DDT has been detected in local assays. To ensure that the goal of elimination is achieved, the implementation of highly-effective transmission-reducing interventions (such as the combined use of IRS and LLINs) for an indefinite period and proper management of malaria cases must remain in place for a long period, taking into account that no vaccine exists to maintain some protective immunity in the island.

At this stage, however, a number of problems are foreseen, which require urgent reviewing of the control methods used:

a) the usage of insecticides, which does not seem to be a sustainable solution, as resistance is a constant and perhaps inevitable threat [14]. This has been documented and reviewed [35,36]. The continuous and regular using of IRS with alphacypermethrin, which is chemically similar to the insecticides used on LLINs, may be a strong selective factor in favor of the development of insecticide resistance [37]. There is, therefore, the need for continuous monitoring for pyrethroid resistance, with susceptibility tests and analysis of genetic mutations in the vector populations, using standardized and approved methods.

b) the reduction of mortality and morbidity due to the five-year control programme in Príncipe, seem to have affected people's perception of the severity of malaria, as they have rejected the continuation of control measures in some villages. Some parents were now treating malaria lightly and this was reflected in the low usage of LLINs, especially for children under five years of age, at present. Prior to this programme, insecticide-treated nets had been distributed and promoted successfully [38]. Educational programmes and a better interaction with the communities are urgently needed as social mobilization measures were not regularly applied. Recently, the Red Cross initiated an intensive programme to advocate using LLINs for malaria prevention, particularly in children and pregnant women, through TV propaganda.

c) re-introduction by travellers is a potential threat. The increase of SPR, by passive case detection, as seen in June 2009, provided an early warning for a possible occurrence of a malaria outbreak, which was prevented by close monitoring of the cases, which seem to have been imported. These malaria hot spots on the island, with recurring finding of new cases, are around the coastal areas with one or two hundred inhabitants each, in typical fishing village settings. Fishermen from São Tomé are known to travel to Príncipe, seasonally, where they temporarily live in simple crude thatched-huts. Active detection should, therefore, be done regularly on these areas and these temporary residents, and all positive malaria cases should be treated promptly and followed up.

Legislation is also needed to assure a prompt response from monitoring systems including the regulation for imported cases from endemic areas. Malaria prevalence is higher on the main and more populated island of São Tome than it is in Príncipe [1], and it is likely to act as a potential source of the malaria hot spots here. Migrants from other West African countries also come to the island, and may carry other diverse strains of malaria parasites, and unless imported malaria is under control, and a good policy on vigilance is set up, the present optimism may be short-lived.

Estimate of financial costs for integrated malaria control programmes in Príncipe was at a range of 7-10 USD per capita per year during the past five years. To sustain such an effective programme in Príncipe, a similar annual budget will be definitely spent for a couple of years at the phase of pre-elimination. In terms of protection from malaria, the cost would be much higher in Príncipe if compared with other countries. In Asia, it was estimated that annual cost per person protected from malaria was at a range of 0.45 - 1.33 USD in 2006 [29]. Príncipe is a very small island with low population even if compared with Zanzibar and Bioko. The cost-effectiveness for such intensive malaria control programmes applied to large areas is always a major concern. The combined malaria control programmes can be successfully carried out in these islands while they might not be generalized, particularly when applied to mainland tropical Africa, with different epidemiology, political and financial settings.

## Conclusions

A five-year integrated control programme has reduced malaria visibly in the island of Príncipe but it remains a public health issue requiring regular monitoring and vigilance. Seasonal malaria outbreaks can occur and worsen if current measures cannot be sustained and evaluated regularly. The establishment of a warning system by early reports and active detection, including the patient's family and migrants, may facilitate the elimination of native malaria from the island and avoid malaria epidemics as it happened in the 80's. Imported malaria cases should be actively searched through a strict control for both flights and ships, particularly those frequently coming from São Tomé island and the continent. Sustaining both vector control interventions and active detection with effective case management must be fully supported by political and financial commitments. At present, malaria elimination seems feasible depending on an active monitoring and vigilance control programme, which requires stronger infrastructure and trained personnel at different levels.

## Competing interests

The authors declare that they have no competing interests.

## Authors' contributions

PWL was responsible for the field work, study coordination, laboratory analysis including PCR confirmation, and data collection. CTL carried out the mosquito cultivation, bioassay, and supervised the field work. HSR contributed to the study design, study coordination, and organized field work. VEdR helped in reviewing the manuscript and discussions. MFS led the conceptual design, study coordination, supervision, data interpretation and manuscript preparation. All authors read and approved the final manuscript.
